# A Facile Inhibitor Screening of Hepatitis C Virus NS3 Protein Using Nanoparticle-Based RNA

**DOI:** 10.3390/bios2040427

**Published:** 2012-10-24

**Authors:** Changhyun Roh

**Affiliations:** Division of Biotechnology, Advanced Radiation Technology Institute (ARTI), Korea Atomic Energy Research Institute (KAERI), 1266, Sinjeong-dong, Jeongeup, Jeonbuk 580-185, Korea; E-Mail: chroh@kaeri.re.kr; Tel.: +82-63-570-3133; Fax: + 82-63-570-3139

**Keywords:** hepatitis C virus, RNA oligonucleotide, quantum dots, inhibitor, screening

## Abstract

Globally, over hundreds of million people are infected with the hepatitis C virus: the global rate of death as a direct result of the hepatitis C virus has increased remarkably. For this reason, the development of efficient drug treatments for the biological effects of the hepatitis C virus is highly necessary. We have previously shown that quantum dots (QDs)-conjugated RNA oligonucleotide can recognize the hepatitis C virus NS3 protein specifically and sensitively. In this study, we elucidated that this biochip can analyze inhibitors to the hepatitis C virus NS3 protein using a nanoparticle-based RNA oligonucleotide. Among the polyphenolic compounds examined, 7,8,4*'*-trihydroxyisoflavone and 6,7,4*'*-trihydroxyisoflavone demonstrated a remarkable inhibition activity on the hepatitis C virus NS3 protein. Both 7,8,4*'*-trihydroxyisoflavone and 6,7,4*'*-trihydroxyisoflavone attenuated the binding affinity in a concentrated manner as evidenced by QDs conjugated RNA oligonucleotide. At a concentration of 0.01 μg·mL^−1^, 7,8,4*'*-trihydroxyisoflavone and 6,7,4*'*-trihydroxyisoflavone showed more than a 30% inhibition activity of a nanoparticle-based RNA oligonucleotide biochip system.

## 1. Introduction

Hepatitis C virus is a small (50 nm in size), enveloped, single-stranded, positive sense RNA virus about 9,600 nucleotides in length encoding approximately 3,010 amino acids [1,2]. Hepatitis C virus NS3 protein activity on the nonstructural proteins of HCV was reported as essential for RNA unwinding, presumably during the viral proliferation and replication [3]. Considering the functional proteins of HCV, the NS3 protein is an important target of HCV therapeutic agent development and is the most attractive target biomarker to capture and monitor. For this reason, the hepatitis C virus NS3 protein is an attractive and crucial target for anti-HCV therapeutic drug discovery. 

Polyphenolic compounds are phytochemicals found in numerous plants and fruits [4,5]. They have been reported to act as antioxidants, free radical scavengers, metal chelators, anti-allergenic, anti-cancer, antioxidant, anti-inflammatory, anti-fungal and contains anti-viral and anti-bacterial agents. In general, these polyphenolic compounds are known to perform medicinal and chemopreventive activities in human health [6,7,8]. In particular, ortho-dihydroxyisoflavones (7,8,4*'*-trihydroxyisoflavone and 6,7,4*'*-trihdyroxyisoflavone) are of growing scientific interest because of their ability to enhance health-related qualities in humans. The hydroxylated compounds at a specific ortho position of isoflavones have potent antioxidant properties that contribute to their cholesterol lowering effect, cardiovascular protection, anti-tumor effect, and anti-carcinogenic properties [9,10]. Quantum dots (QDs), which are semiconductor nanocrystals made up of semiconductor materials with a CdSe/ZnS core-shell, have attracted remarkable attention in the fields of nanotechnology and biotechnology, especially in fluorescence-based biological imaging applications [11,12]. In this study, we report a novel approach for the inhibitor screening of the hepatitis C virus NS3 protein using a QDs-conjugated oligonucleotide system on an imaging biochip. We elucidated an inhibitor of the hepatitis C virus NS3 protein, identified through a high-throughput screening strategy, using an optical nanoparticle-based RNA oligonucleotide. To the best of our knowledge, this is the first report on the inhibition effects of 7,8,4*'*-trihydroxyisoflavone and 6,7,4*'*-trihydroxyisoflavone on hepatitis C virus NS3 protein using a nanoparticle-based RNA oligonucleotide platform.

## 2. Experimental Section

### 2.1. Chemicals

EDC (N-(3-dimethylaminopropyl)-N*'*-ethylcarbodiimide hydrochloride) and kanamycin were purchased from Sigma-Aldrich Chemical Co. (St. Louis, MO, USA). Quantum dots (QDs605) were purchased from Invitrogen Corporation (Carlsbad, CA, USA). Prolinker^TM^-terminated glass slide were purchased from Proteogen (Seoul, Korea). All other chemicals were of the highest grade. 

### 2.2. Conjugation of Quantum Dots and RNA Oligonucleotide

An amine group with a terminal modification of the NS3 RNA oligonucleotide was synthesized by BIONEER Co. Ltd. (Seoul, Korea) and carboxyl-terminated QDs605 was purchased from Invitrogen (Carlsbad, CA, USA). The amino group of the NS3 RNA oligonucleotide (H2N-5*'*-GCAGUAGUGUAUAGGC-3*'*) was first covalently conjugated onto the surface of the carboxyl-terminated QDs (10 pM, 1.25 μL). That is, 10 pM of QDs were conjugated with 400 pM of oligonucleotide with 1 μL of 40 nM EDC, to activate amide bond formation to produce QDs-conjugated oligonucleotide (QDs-NS5B oligonucleotide) at a QDs:RNA oligonucleotide molar ratio of 1:40 for 1 h at room temperature. Thereafter, the QDs-oligonucleotide conjugate was collected using centrifugal filtration at 15,000 rpm for 30 min, followed by several washing steps with a Tris buffer (50 mM Tris-HCl pH 7.4, 5 mM KCl, 100 mM NaCl, 1 mM MgCl_2_, and 0.1% NaN_3_). After centrifugal filtration and washing, a pellet of the QDs-conjugated RNA oligonucleotide was dispersed through a brief sonication (22 kHz, amplitude of 12 μm, and sonication time of 120 s) using a sonic dismembrator (Model F60 Sonic Dismembrator; Fisher Scientific, Fair Lawn, NJ, USA).

### 2.3. Optical Assay in a Confocal Laser-Scanning Microscope

The recombinant viral protein NS3 was immobilized directly onto the functional ProLinker-terminated surface. For the inhibition activity, the QDs-conjugated RNA oligonucleotide and inhibitor were facilitated by spotting on an immobilized HCV viral protein glass chip. After incubation for 1 h at 25 °C, the glass chip was then washed three times with the phosphate buffer (pH 7.2) for 1 min. The glass chip was analyzed using a confocal laser-scanning microscope, an LSM 510 META (Carl Zeiss, Jena, Germany). The signal intensity was determined using software for the LSM510 (LSM Image Browser). A histogram of the intensity was obtained from the region of the spotted chip. The value of the signal intensity was obtained by calculating and expressing it as the mean intensity.

## 3. Results and Discussion

### 3.1. Scheme for Inhibitor Screening of Viral Protein NS3 on Biochip

**Figure 1:**
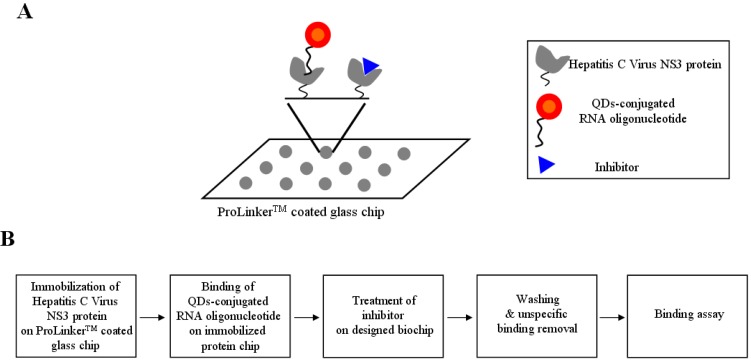
A representative scheme for the inhibitor screening of hepatitis c virus NS3protein using nanoparticle-conjugated RNA oligonucleotide on biochip.

For the inhibitor screening of the hepatitis C virus NS3 protein, we designed the QDs-conjugated specific RNA oligonucleotide for specific hepatitis C virus NS3 protein targeting: first, the hepatitis C virus NS3 protein (1 μL) was immobilized on a glass chip; second, QDs-conjugated RNA oligonucleotide conjugates (1 μL) were bound on an immobilized chip; third, inhibitor treatment was performed on the conjugated RNA oligonucleotide and hepatitis C virus NS3 protein; fourth, washing and unspecific binding removal was done; fifth, detection was achieved to show directly the specific recognition of the inhibition effect of hepatitis C virus NS3 protein on the biochip. The schematic design of the inhibitor screening for effective monitoring of hepatitis C virus NS3 protein is illustrated in Figure 1. To accomplish the feasibility of targeting and imaging, we used QD605 conjugates having an RNA oligonucleotide for hepatitis C virus NS3 protein with an emission wavelength as the optical imaging probe.

### 3.2. Expression and Purification of Viral Protein NS3

We expressed the recombinant HCV NS3 in the *E. coli* expression system and purified it by Ni-NTA affinity chromatography. In the purification step, the protein was eluted in 1 mL fraction with a 500 mM imidazole buffer. To verify the purity and homogeneity of the eluted NS3, aliquots of eluted fractions were analyzed by Coomassie blue staining of the SDS-PAGE prior to pooling the fractions. Eluates with the highest purity of 95% were pooled, dialyzed and stored with 50% glycerol in aliquots at −80 °C. The HCV NS3 protein was purified by a single chromatography step on a Ni^2+^ affinity column. The C-terminally his-tagged HCV NS3 was visualized with a molecular mass of approximately 68 kDa on a SDS-PAGE (data not shown).

### 3.3. Inhibitory Effect of Viral Protein NS3

**Figure 2:**
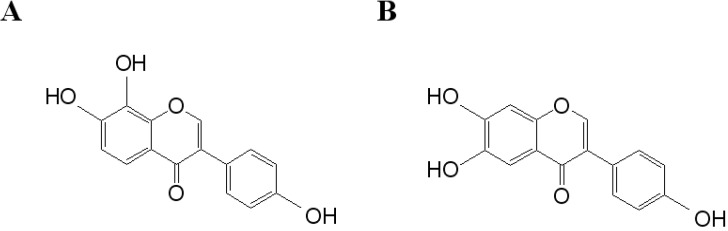
Chemical structures of (**A**) 7,8,4*'*-trihydroxyisoflavone and (**B**) 6,7,4*'*-trihydroxyisoflavone.

**Figure 3:**
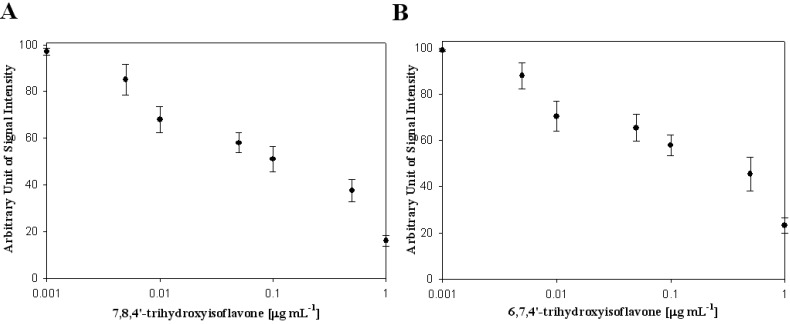
Inhibitory effect of (**A**) 7,8,4*'*-trihydroxyisoflavone and (**B**) 6,7,4*'*-trihydroxyisoflavone on viral protein NS3.

In Table 1, the effects of polyphenolic compounds on the inhibition of the hepatitis C virus NS3 protein used in this study are presented. Among the polyphenolic compounds screened, 7,8,4*'*-trihydroxyisoflavone and 6,7,4*'*-trihydroxyisoflavone showed high anti-viral activity. The chemical structures of 7,8,4*'*-trihydroxyisoflavone and 6,7,4*'*-trihydroxyisoflavone are shown in Figure 2. Figure 3 shows that both 7,8,4*'*-trihydroxyisoflavone and 6,7,4*'*-trihydroxyisoflavone had high inhibition activity in a concentrated manner against the hepatitis C virus NS3 protein. At a concentration of 0.01 μg·mL^–1^, 7,8,4*'*-trihydroxyisoflavone and 6,7,4*'*-trihydroxyisoflavone showed more than 30% inhibition activity of a QDs-RNA oligonucleotide biochip platform. As shown in Figure 3(A,B), 7,8,4*'*-trihydroxyisoflavone and 6,7,4*'*-trihydroxyisoflavone showed a similar pattern when comparing the concentration-dependent anti-viral activity. The half-maximal inhibitory concentration (IC_50_) values of 7,8,4*'*-trihydroxyisoflavone and 6,7,4*'*-trihydroxyisoflavone were found to be approximately 0.1 μg·mL^–1^ and 0.5 μg·mL^–1^, respectively (Figure 3(A,B)). Other polyphenolic compounds for the inhibition of the hepatitis C virus NS3 protein on the nanoparticle-based RNA oligonucleotide biochip system were detected as nearly similar to the background signal, due to the high affinity with the QDs-conjugated aptamer-hepatitis C virus NS3 protein (data not shown). To perform a high-throughput screening of the inhibitors, it would be efficient to be able to measure the anti-viral activity from optical images of a biochip containing multiple reaction compounds. The inhibition of the anti-viral activity from the hepatitis C virus NS3 protein was clearly illustrated and dose dependency was distinctly observable in the optical images. We demonstrated the specific interaction for inhibitor screening between RNA aptamer and HCV NS3 viral protein on biochip platform. We discovered a novel function of 7,8,4*'*-trihydroxyisoflavone and 6,7,4*'*-trihydroxyisoflavone as an antiviral agent. The discovery of antiviral drugs has been of considerable interest in developing efficient and sensitive methods for high-throughput screening in medicine. Furthermore, this proposed method can be considered a real-time monitoring method for inhibitor screening of HCV viral protein and is expected to be applicable to other types of diseases.

**Table 1:** The effects of polyphenolic compounds on the inhibition of viral protein NS3.

Compounds	Inhibition Activity	Compounds	Inhibition Activity
Daidzein	-	Quercetin	-
Genistein	-	Acacetin	-
7,8,4*'*-trihydroxyisoflavone	+	Apigenin	-
6,7,4*'*-trihydroxyisoflavone	+	Baicalein	-
Glycitein	-	Hesperidin	-
Kaempferol	-	Morin	-
Luteolin	-	Rutin	-
Myricetin	-	Naringin	-
Silibinin	-	Naringenin	-
Silymarin	-	(-)-Catechin	-
Orientin	-	(-)-Catechin gallate	-
Oroxylin A	-	(-)-Gallocatechin gallate	-

+: It means this has inhibition activity on hepatitis C virus NS3 protein; -: It means this has no inhibition activity on hepatitis C virus NS3 protein.

## 4. Conclusions

In this study, we demonstrated inhibitor screening on a designed biochip platform using a nanoparticle based RNA oligonucleotide. We discovered a novel function of 7,8,4*'*-trihydroxyisoflavone and 6,7,4*'*-trihydroxyisoflavone as an antiviral agent. The discovery of antiviral drugs has been of considerable interest in developing efficient and effective methods for HTS screening in medicine. Our main goal in this study was to demonstrate a proof-of-concept that viral protein can be inhibited and detected with remarkable sensitivity and simplicity. 
